# Multi-faceted reactivity of *N*-fluorobenzenesulfonimide (NFSI) under mechanochemical conditions: fluorination, fluorodemethylation, sulfonylation, and amidation reactions

**DOI:** 10.3762/bjoc.18.20

**Published:** 2022-02-07

**Authors:** José G Hernández, Karen J Ardila-Fierro, Dajana Barišić, Hervé Geneste

**Affiliations:** 1 Division of Physical Chemistry, Ruđer Bošković Institute, Bijenička c. 54, 10000 Zagreb, Croatia; 2 current address Instituto de Química, Facultad de Ciencias Exactas y Naturales, Universidad de Antioquia, Calle 70 No 52-21, Medellín, Colombia; 3 Division of Materials Chemistry, Ruđer Bošković Institute, Bijenička c. 54, 10000 Zagreb, Croatia; 4 AbbVie Deutschland GmbH & Co. KG, Neuroscience Research, D-67008 Ludwigshafen, Germany

**Keywords:** amidation, ball mill, fluorination, in situ monitoring, mechanochemistry, NFSI, Raman monitoring, sulfonylation

## Abstract

In the search for versatile reagents compatible with mechanochemical techniques, in this work we studied the reactivity of *N*-fluorobenzenesulfonimide (NFSI) by ball milling. We corroborated that, by mechanochemistry, NFSI can engage in a variety of reactions such as fluorinations, fluorodemethylations, sulfonylations, and amidations. In comparison to the protocols reported in solution, the mechanochemical reactions were accomplished in the absence of solvents, in short reaction times, and in yields comparable to or higher than their solvent-based counterparts.

## Introduction

Mechanosynthesis of organic molecules and materials using mechanochemical techniques such as ball milling, extrusion, grinding, etc. [1–3] have enabled the development of known and new chemical transformations in a more sustainable fashion [4]. Commonly, mechanochemical reactions by ball milling involve the mechanical treatment of at least one solid reagent in the presence of other solid, liquid or gaseous reaction partners or additives [5–6]. Due to the particular reaction conditions in which mechanochemical reactions by milling are carried out, reagents need to exhibit stability under environments of mechanical stress, while at the same time enough reactivity to engage in chemical transformations. In the search for solid reagents compatible with mechanochemical techniques, we became interested in evaluating the behavior of *N*-fluorobenzenesulfonimide (NFSI) under ball-milling conditions. NFSI is a colorless crystalline powder (mp 114–116 °C), bench-stable, and an easy-to-handle reagent, which, due to its commercial availability, has been extensively used as a fluorinating agent in solution [7–9]. Additionally, NFSI has also been explored as an oxidant, amidation reagent [9–11], and phenylsulfonyl group transfer reagent [12–13].

**Scheme 1:**
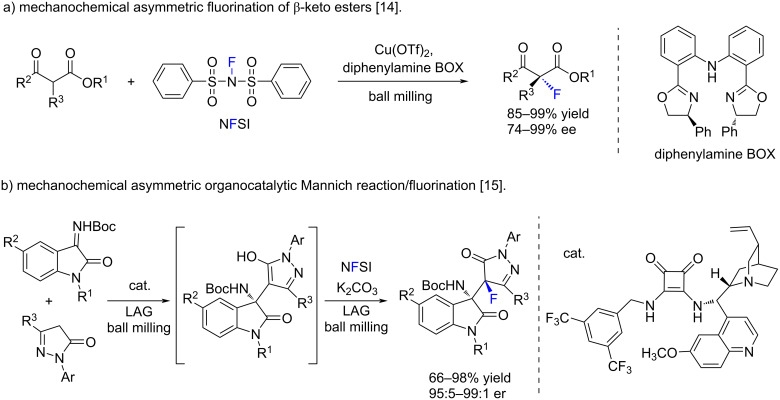
Examples of mechanochemical reactions using NFSI.

In the field of mechanochemistry, the usefulness of *N*-fluorobenzenesulfonimide has been exemplified in the asymmetric fluorination of β-keto esters (Scheme 1a) [14], and in diastereoselective fluorinations (Scheme 1b) [15], which complemented mechanochemical fluorinations carried out with other reagents, such as AgF [16], 1-chloromethyl-4-fluoro-1,4-diazoniabicyclo[2.2.2]octane bis(tetrafluoroborate) (Selectfluor^®^) [17–20], among other fluorinating reagents [21].

However, as shown above, examples using NFSI by mechanochemistry are scarce and they have mostly been focused on the fluorinations of enolizable substrates. These considerations led us to explore the behavior of NFSI in fluorinations of activated arenes under ball-milling conditions towards an eventual implementation of mechanochemistry in late-stage C–H functionalizations [22–23]. In particular, efficient fluorination protocols are long sought after in several areas of science, including medicinal chemistry [24]. Next to fluorination, in this work, we also have investigated NFSI as a source for mechanochemical sulfonylation of imidazoles and amidation reactions.

## Results and Discussion

**Scheme 2:**
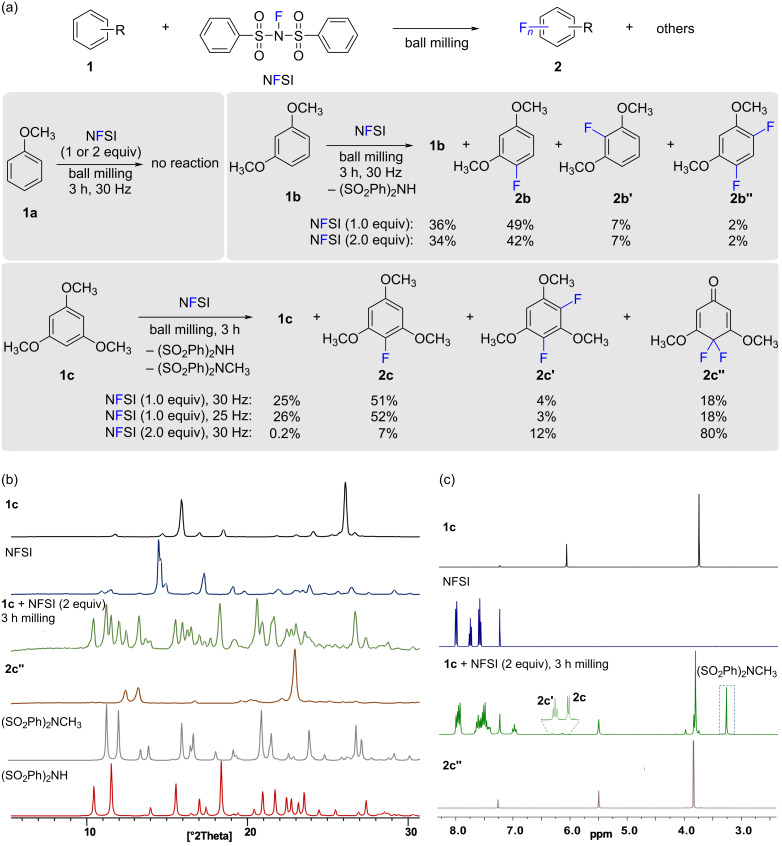
Mechanochemical fluorination of arenes **1** with NFSI. (a) Product distributions and reaction conditions: Arenes **1** (0.148 mmol) were milled with NFSI (1.0–2.0 equiv) in a 2 mL Eppendorf tube with four ZrO_2_ milling balls (350 mg in total mass) at 25–30 Hz for 3 h. The yield of the products was determined by NMR analysis with 4-fluoroacetanilide as the internal standard. Similar product distribution was obtained when a mixture of **1c** (0.59 mol) and NFSI (1.18 mmol) was milled in a stainless steel milling jar (15 mL of internal volume) using one milling ball (4.0 g) of the same material: **2c** yield = 7%; **2c'** yield = 16%; **2c''** yield = 79%; (SO_2_Ph)_2_NCH_3_ yield = 82%. (b) Powder X-ray diffraction (PXRD) patterns measured for the reactants and simulated PXRD patterns for the published (SO_2_Ph)_2_NCH_3_ (CCDC ZAJBIZ) and (SO_2_Ph)_2_NH (CCDC BSULFA). (c) ^1^H NMR analysis of the reaction mixture of the milling of **1c** and NFSI (2.0 equiv) at 30 Hz for 3 h.

Previous reports in the absence of solvent have shown that NFSI promotes aromatic fluorination at temperatures between 80 °C and 105 °C [25]. To commence, we focused on the reaction between arenes **1a**–**c** and NFSI by ball milling in the absence of external heating (Scheme 2a). To conduct a high-throughput screening we initially carried out the milling experiments in Eppendorf vials before using standard milling jars made of stainless steel or poly(methyl methacrylate) (PMMA). This simple approach accelerated the optimization of the milling and reaction parameters [26]. From a sustainable point of view, experimenting in small scale could prevent waste production and increase safety. However, miniaturization of mechanochemical reactions could also be an alternative to working with precious or expensive reagents and to facilitate monitoring of the reactions [27].

Under such reaction conditions anisole (**1a**) did not undergo fluorination even in the presence of two equivalents of NFSI. However, more activated 1,3-dimethoxybenzene (**1b**) gave a mixture of principally monofluorinated products **2b** and **2b'**. Reacting 1,3,5-trimethoxybenzene (**1c**) and NFSI (1.0 equiv) also gave preferentially monofluorinated product **2c** in 51% yield. Analysis of the milled mixture by ^1^H and ^19^F NMR spectroscopy also revealed the presence of difluorinated products **2c'** and **2c''**. The product composition of the reaction of **1c** with NFSI (1.0 equiv) remained unchanged at lower milling speeds (25 Hz vs 30 Hz), but the use of two equivalents of NFSI afforded 4,4-difluoro-3,5-dimethoxy-2,5-cyclohexadienone (**2c''**) as the major product in 80% yield (Scheme 2a). Mechanistically, formation of **2c''** from 1,3,5-trimethoxybenzene (**1c**) requires a fluorodemethylation pathway to be operational under the ball-milling conditions, for example through the reaction of product **2c** with the second equivalent of NFSI. In solution, 1,3,5-trimethoxybenzene (**1c**) has been reported to undergo fluorodemethylation when reacted with Selectfluor^®^, however the authors mentioned that “the fate of the methyl group lost in the conversion” of **2c** to **2c''** “remain[ed] obscure” [28]. In our case, we anticipated that formation of **2c''** could be accompanied by concomitant formation of (PhSO_2_)_2_NH and (PhSO_2_)_2_NCH_3_ derived from NFSI and **1c**. For the analysis of the reaction mixture we selected powder X-ray diffraction (PXRD), a rapid analytical technique that has proven useful for the structural characterization of crystalline organic solids and which requires minimal sample preparation [29]. Pleasingly, analysis of the milled mixture (**1c** + NFSI) by PXRD evidenced the existence of diffraction reflections corresponding to crystalline (PhSO_2_)_2_NH, (PhSO_2_)_2_NCH_3_, and product **2c''** (Scheme 2b) [30]. Additionally, ^1^H NMR spectroscopy confirmed the presence of (PhSO_2_)_2_NCH_3_ in the reaction mixture after the milling process (Scheme 2c) [31], in yields that matched the ones for **2c''** (Scheme 2a).

**Figure 1:**
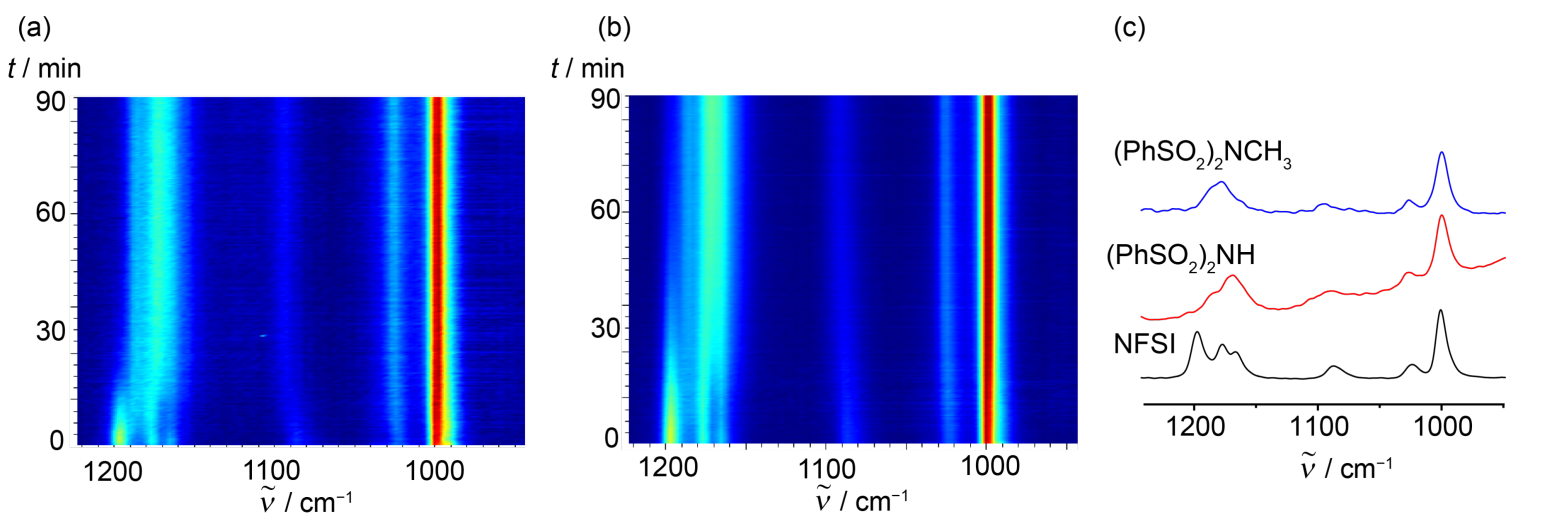
Time-resolved 2D plots of the mechanochemical reaction of: (a) **1c** (0.59 mmol), NFSI (1.0 equiv), and SiO_2_ (300 mg). (b) **1c** (0.59 mmol), NFSI (2.0 equiv), and SiO_2_ (300 mg). Both reactions were carried out at 30 Hz using milling jars made of PMMA (15 mL of internal volume) with one ZrO_2_ milling ball (3.4 g). (c) Raman spectra of pure NFSI, (PhSO_2_)_2_NH, and (PhSO_2_)_2_NCH_3_.

To get some insights into the mechanochemical reaction of **1c** with NFSI we have performed in situ reaction monitoring of the milling process by Raman spectroscopy [32–33]. In an experiment milling **1c** with NFSI (1 equiv) we observed the consumption of NFSI after ca. 30 min of milling as evidenced by a reduction in the intensity of the band at 1197 cm^−1^ of NFSI (Figure S3 in Supporting Information File 1). However, the very strong bands around 998 cm^−1^ (in-plane bending; phenyl ring), 1177 cm^−1^ (stretching; SO_2_), and 1583 cm^−1^ (stretching; phenyl ring) of NFSI and byproducts [(PhSO_2_)_2_NH [34], and (PhSO_2_)_2_NCH_3_], prevented the observation of the less Raman active fluorinated products **2c** and **2c''**. Even though **1c** and NFSI are solids (mp**_1c_** = 50–53; mp_NFSI_ = 114–116 °C), rheological changes of the reaction mixture upon milling and formation of liquid **2c** rendered a sticky reaction mixture, which affected the quality of the Raman monitoring (Figures S3 and S4 in Supporting Information File 1). To mitigate this, the milling experiment was repeated using silica gel (SiO_2_) as a milling auxiliary. The use of SiO_2_ did not affect significantly the product composition of the reaction as determined by NMR analysis of an independent experiment milling **1c** and NFSI (2.0 equiv) at 30 Hz for 3 h. This reaction gave a mixture of **2c''**, **2c'**, and **2c** in a ratio of 79:16:5 vs a ratio of 80:12:7 in the absence of SiO_2_ (Scheme 2a). Moreover, the presence of SiO_2_ improved the absorption of reactants and rendered a reaction mixture physically more appropriate for the milling process, which in turn enabled a better monitoring of the transformation and favored the reaction to be completed in shorter milling times (Figure 1a and Figure S5 in Supporting Information File 1). The mechanochemical reaction of **1c** with NFSI (2 equiv) was also monitored revealing that the consumption of NFSI required ca. 30 min of milling (Figure 1b and Figure S5 in Supporting Information File 1).

Other substrates such as naphthalene and *N*-Boc-aniline proved unreactive under the milling conditions with NFSI. However, the more activated arene 2-naphthol underwent double fluorination affording 1,1-difluoronaphthalen-2(1*H*)-one as the major product (i.e., 29% yield using 1.0 equiv of NFSI and 51% yield using 2.0 equiv of NFSI after 3 h of milling at 30 Hz).

**Scheme 3:**
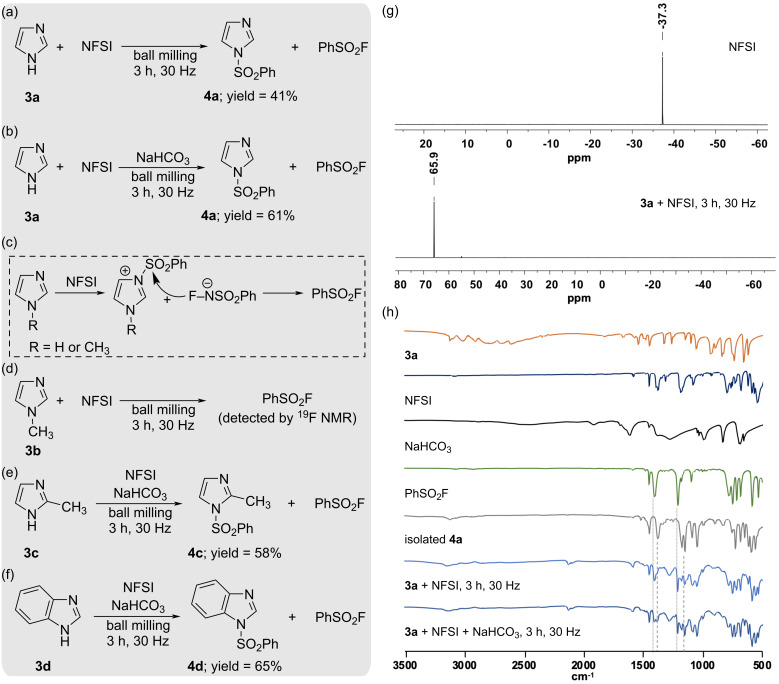
(a–f) Reactions of substrates **3** with NFSI. Reaction conditions: Substrates **3** (0.734 mmol) were milled with NFSI (1.0 equiv) and NaHCO_3_ (0.5 equiv) in a stainless steel milling jar (15 mL of internal volume) using one stainless steel milling ball (4.0 g). (g) ^19^F NMR spectra for NFSI (top) and **3a** + NFSI milled (bottom). (h) Analysis by ATR-FTIR spectroscopy of the reaction of **3a** with NFSI.

After having studied the ability of NFSI to participate in fluorination and fluorodemethylation reactions, we evaluated the capacity of NFSI to act as a sulfonyl source. For this, we reacted a mixture of NFSI and imidazole (**3a**) by ball milling. Analysis by NMR spectroscopy of the crude reaction mixture showed that 1-(benzenesulfonyl)imidazole (**4a**) had been formed in 41% yield (Scheme 3a).

Complementarily, ^19^F NMR spectroscopy of the crude reaction mixture evidenced a distinctive peak at 65.8 ppm in the ^19^F NMR spectrum (Scheme 3g), which was assigned to phenylsulfonyl fluoride (PhSO_2_F) [35], a byproduct often obtained in reactions with NFSI [36–37]. Trying to improve the rheology of the reaction mixture and to increase the basicity of the medium, we milled **3a** and NFSI in the presence of NaHCO_3_, which had a positive effect affording product **4a** in 61% yield (Scheme 3b), which is significantly higher than the 46% yield reported in CH_3_CN after 12 h at 80 °C [38]. The generation of **4a** upon milling was demonstrated after immediate analysis of the milled sample by ATR-FTIR spectroscopy (Scheme 3h).

Formation of **4a** could have occurred from the direct reaction of the N–H nitrogen of **3a** with NFSI, which would agree with the propensity for NFSI to react with some hard oxygen and nitrogen nucleophiles at the sulfur atom instead of at the fluorine atom [39–40]. Similarly, NFSI has also been reported to act as a transfer of the sulfonyl moiety from NFSI to carbon centers [12–13]. Alternatively, **4a** could have been formed from the reaction of imidazole (**3a**) with the in situ formed PhSO_2_F. To better understand the formation of PhSO_2_F during the milling of NFSI with **3a**, we reacted its N-methylated derivative **3b**, a substrate unable to undergo the sulfonylation pathway with NFSI. We hypothesized that PhSO_2_F could have been generated after an initial reaction of the nitrogen with the lone electron pair in imidazole at the sulfonyl group of the NFSI (Scheme 3c), mimicking the reactivity of pyridine derivatives with NFSI, which are known to generate phenylsulfonyl fluoride via a transient generation of *N*-sulfonylpyridinium salts [37].

Analysis by ^19^F NMR spectroscopy of the crude reaction mixture of **3b** and NFSI revealed the presence of PhSO_2_F (Scheme 3d), thus confirming the capacity of the nitrogen with the lone electron pair in **3b**, and probably in **3a**, to react with NFSI at the sulfonyl group to facilitate the formation of phenylsulfonyl fluoride (Scheme 3c). Other imidazole derivatives such as 2-methylimidazole (**3c**) and benzimidazole (**3d**) also underwent sulfonylation affording products **4c** and **4d** in 58% yield and 65% yield, respectively (Scheme 3e and 3f).

**Scheme 4:**
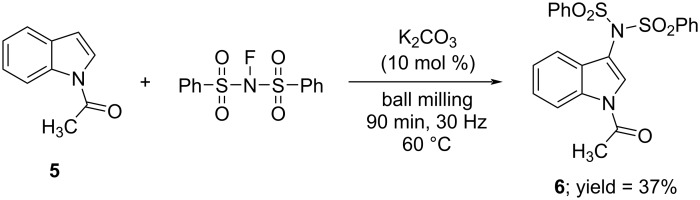
Regioselective C-3 mechanochemical amidation of **5** with NFSI.

Finally, to corroborate that other known chemical pathways for NFSI, including amidation reactions, could be accessible under mechanochemical conditions we studied the reaction of 1-acetylindole (**5**) with NFSI. In solution (i.e., dichloroethane, 60 °C, 24 h, Ar atmosphere), **5** undergoes regioselective C-3 amidation with NFSI using catalytic amounts of K_2_CO_3_ [41]. An initial attempt to carry out the reaction by ball milling **5**, NFSI (2 equiv), and K_2_CO_3_ (10 mol %) for 3 h afforded only traces of the aminated product **6**. To assist the metal-free amidation of the aromatic C–H bond in **5**, we repeated the milling experiment at 40 °C using a heat gun to increase the temperature of the milling jar (see Supporting Information File 1, Figure S2) [42], which gave a mixture of **5** and **6** (ratio 85:15). The same experiment at 60 °C led to the full consumption of **5** after 1.5 h of milling and product **6** could be isolated in moderate 37% yield (Scheme 4). Formation of **6** in the absence of external heating was also possible after lengthening the milling time to 16 h, such an experiment afforded a mixture of **5** and **6** (ratio 60:40). In comparison, in dichloroethane at 40 °C the reaction of **5** and NFSI only afforded amidated product **6** in 4% after 24 h of reaction [41].

## Conclusion

In this study we evaluated the multifaceted reactivity of NFSI under mechanochemical conditions. We observed that NFSI was compatible with the ball-milling reaction conditions. Additionally, we corroborated that, by mechanochemistry, NFSI can engage in a variety of reactions known in solution such as fluorinations, fluorodemethylations, sulfonylations, and amidations. These transformations could be accomplished in short milling times in the absence of solvent. Being a crystalline material [43] and a Raman and IR active molecule [44–45], NFSI enabled the monitoring of the reactions by ex situ PXRD and IR spectroscopy, as well as by in situ Raman spectroscopy. Such a monitoring enabled us to understand background reactions such as the fluorodemethylation pathway underwent by 1,3,5-trimethoxybenzene (**1c**) when reacted with NFSI, which was found to procced via initial formation of monofluorinated product **2c**. In general, NFSI could participate in the chemical transformations by ball milling without the need for external heating, however, the amidation of 1-acetylindole (**5**) was found to proceed more efficiently under simultaneous thermal milling conditions. Altogether, the results of this work expand the applicability of NFSI by mechanochemistry beyond fluorination reactions of enolizable substrates and might facilitate the application of NFSI in new reactions by ball milling in the future. Ongoing work on the development of similar mechanochemical reactions using NFSI are being investigated in our laboratories.

## Supporting Information

File 1 Experimental details, characterization data and copies of spectra.
