# Supplementary material for: Multi-faceted reactivity of N-fluorobenzenesulfonimide (NFSI) under mechanochemical conditions: fluorination, fluorodemethylation, sulfonylation, and amidation reactions
Source: Beilstein J Org Chem. 2022 Feb 7;18:182–9. doi: 10.3762/bjoc.18.20 (PMC8848345; doi:10.3762/bjoc.18.20)
Supplement: File 1 — Experimental details, characterization data and copies of spectra. [file Beilstein_J_Org_Chem-18-182-s001.pdf]

BEILSTEIN JOURNAL OF ORGANIC CHEMISTRY

Supporting Information

for

Multi-faceted reactivity of *N*-fluorobenzenesulfonimide (NFSI) under mechanochemical conditions: fluorination, fluorodemethylation, sulfonylation, and amidation reactions

José G. Hernández, Karen J. Ardila-Fierro, Dajana Barišić and Hervé Geneste

Beilstein J. Org. Chem. **2022,** *18,* 182–189. doi:10.3762/bjoc.18.20

Experimental details, characterization data and copies of spectra

License and Terms: This is a supporting information file under the terms of the Creative Commons Attribution License (https://creativecommons.org/licenses/by/4.0). Please note that the reuse, redistribution and reproduction in particular requires that the author(s) and source are credited and that individual graphics may be subject to special legal provisions.
The license is subject to the *Beilstein Journal of Organic Chemistry* terms and conditions: (https://www.beilstein-journals.org/bjoc/terms)

Table of contents

1.0 Chemicals S1
1.1 Milling equipment S1
1.2 Nuclear magnetic resonance (NMR) spectroscopy........ S4
1.3 Powder X-ray diffraction (PXRD) S4
1.4 In-situ monitoring by Raman spectroscopy........ S4
2.0 Mechanochemical reactions S5
3.0 Characterization of the products S6
4.0 Copies of the NMR spectra S8
5.0 References........ S15

1.0 Chemicals

N-Fluorobenzenesulfonimide (98% purity, Carbolution), anisole (99% purity, Fluka), 1,3-dimethoxybenzene (98% purity, Alfa Aesar), 1,3,5-trimethoxybenzene (>99% purity, Aldrich), imidazole (99% purity, Alfa Aesar), 2-methylimidazole (99% purity, Alfa Aesar), and benzimidazole (99% purity, Alfa Aesar) were used as received from the commercial supplier. 1-Acetylindole was prepared according the literature [1].

1.1 Milling equipment

Mechanochemical reactions were carried out using mixer mills (InSolido Technologies, Croatia, Zagreb). Screening ball-milling experiments were carried out in 2 mL round-bottomed Eppendorf tubes with four ZrO_2 balls (total mass 340 mg) (Figure S1). Other milling experiments were carried out using milling jars made of poly(methyl)methacrylate (PMMA) (15 mL in internal volume) with one milling ball made of Zr_2O weighing 3.4 g, or in milling jars made of stainless steel (15 mL in internal volume) with one milling ball of the same material weighing 4.0 g.

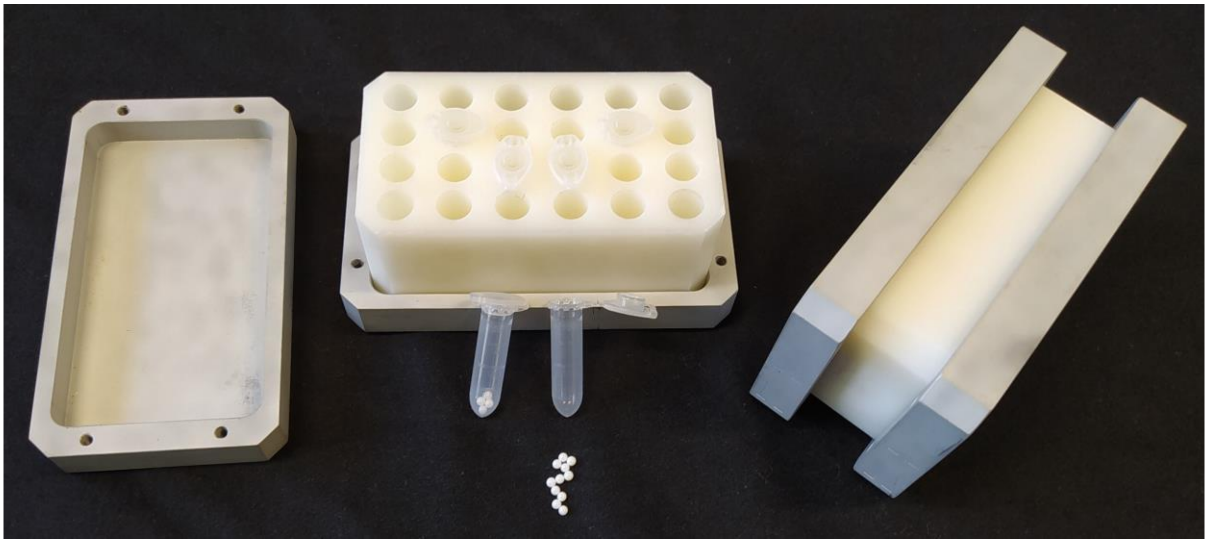

Figure S1: Eppendorf tubes and setup used for the screening milling experiments.

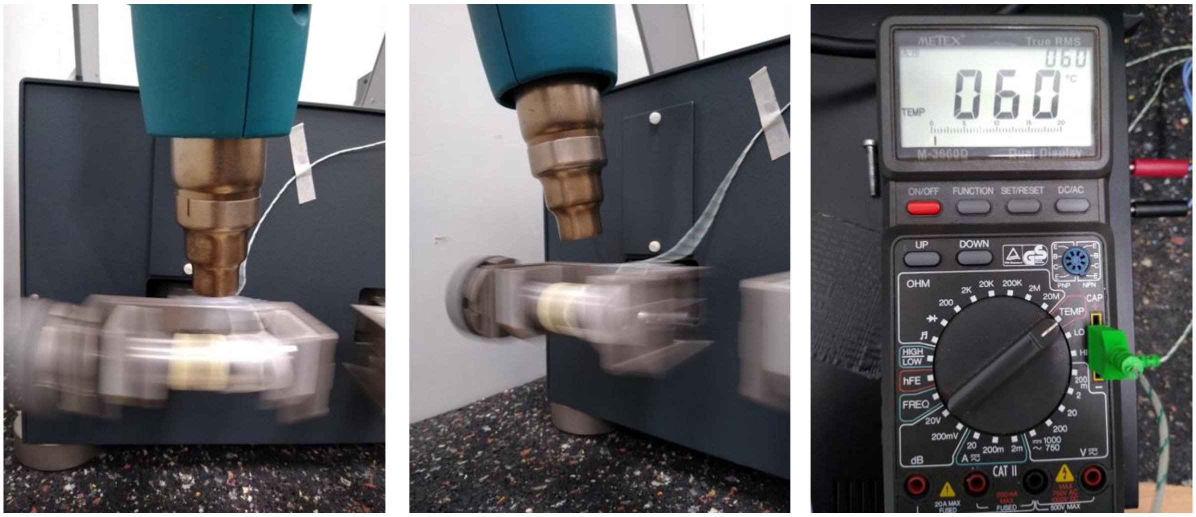

Figure S2: Setup for thermally assisted milling experiments using a heat gun (Makita HG651C) and a digital multimeter (M-3660D) for temperature measurements onthe outer surface of the milling jar.

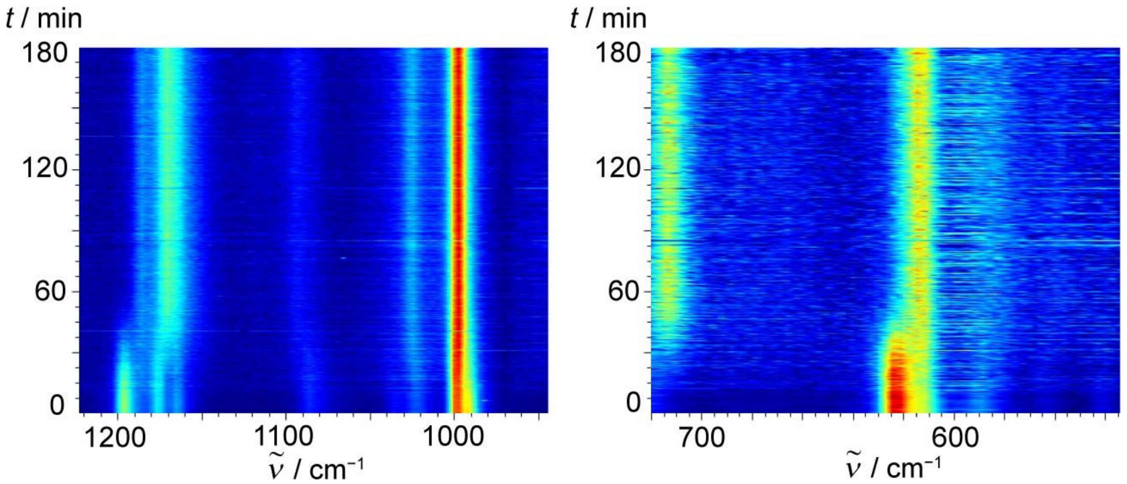

Figure S3: Time-resolved 2D plot of the mechanochemical reaction of **1c** (0.59 mmol) and NFSI (1.0 equiv) at 30 Hz using milling jars made of PMMA (15 mL of internal volume) with one ZrO_2 milling ball (3.4 g).

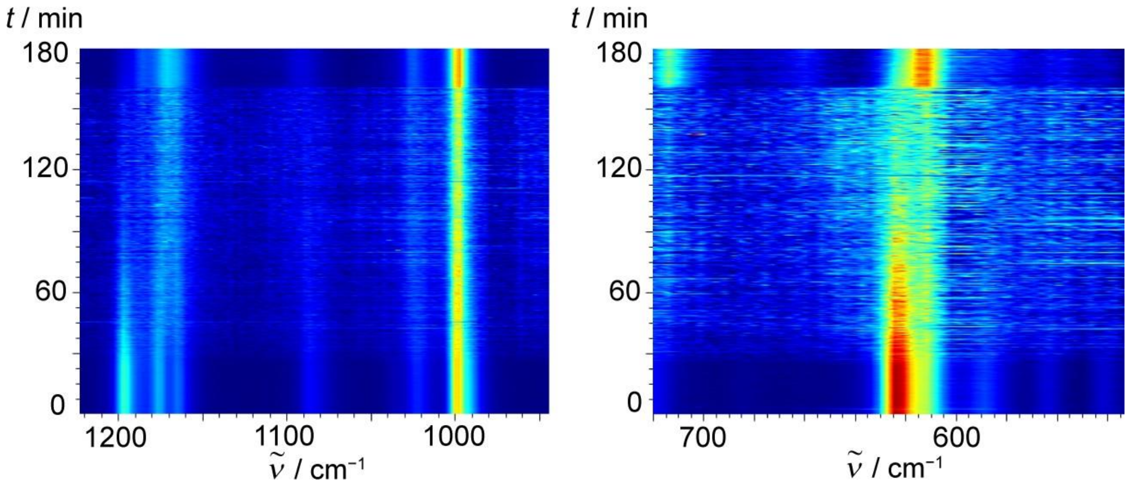

Figure S4: Time-resolved 2D plot of the mechanochemical reaction of **1c** (0.59 mmol) and NFSI (2.0 equiv) at 30 Hz using milling jars made of PMMA (15 mL of internal volume) with one ZrO_2 milling ball (3.4 g).

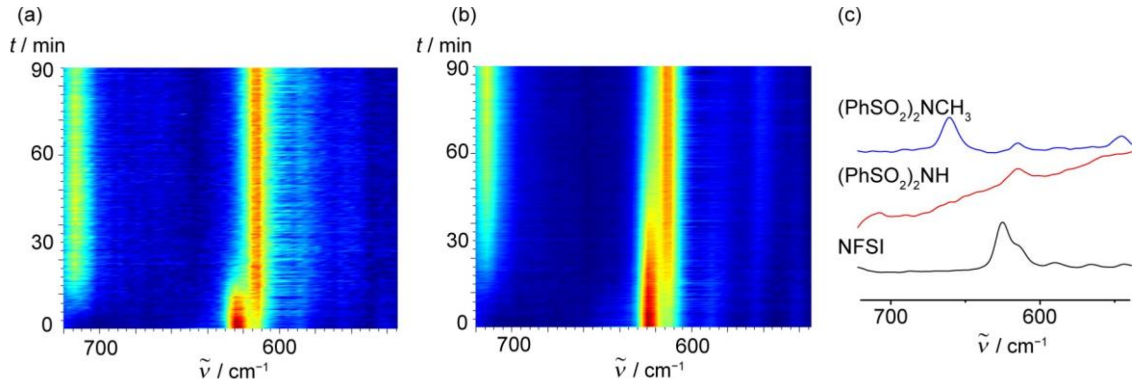

Figure S5: Time-resolved 2D plots of the mechanochemical reaction of: (a) **1c** (0.59 mmol), NFSI (1.0 equiv), and SiO_2 (300 mg). (b) **1c** (0.59 mmol), NFSI (2.0 equiv), and SiO_2 (300 mg). Both reactions were carried out at 30 Hz using milling jars made of PMMA (15 mL of internal volume) with one ZrO_2 milling ball (3.4 g). (c) Raman spectra of pure NFSI, $(PhSO_2)_2NH$, and $(PhSO_2)_2NCH_3$.

1.2 Nuclear magnetic resonance (NMR) spectroscopy

NMR spectra were recorded on a VNMRS 600 spectrometer. Proton and carbon chemical shifts are reported in parts per million and are calibrated using the residual non-deuterated solvent signal ($CDCl_3$) as an internal reference. Fluorine NMR spectra were recorded on a Bruker Avance III HD 600 MHz Ascend spectrometer.

1.3 Powder X-ray diffraction (PXRD)

PXRD patterns were collected on a PanAlytical Aeris diffractometer (CuK_α radiation and Ni filter) in Bragg-Brentano geometry using a zero-background sample holder.

1.4 In-situ monitoring by Raman spectroscopy

Laboratory in-situ Raman monitoring was performed using portable Raman system with a PDLD (now Necsel) BlueBox laser source with the excitation wavelength of 785 nm equipped with B&W-Tek fiber optic Raman BAC102 probe and coupled with

one OceanOptics Maya2000Pro spectrometer (with resolutions of 1 cm^{-1} or 3.5 cm^{-1}). The probe was positioned about 1 cm under a reaction vessel on a moving stand, and laser was focused 1 mm inside of the inner vessel wall. Time-resolved in- situ Raman spectra were collected in an automated fashion using an in-house code in MATLAB. Subtraction of vessel contribution to Raman spectra was described elsewhere [2].

2.0 Mechanochemical reactions

2.1 Mechanochemical reaction of 1c with NFSI

A mixture of **1c** (0.148 mmol) and NFSI (1.0–2.0 equiv) was milled in a 2 mL Eppendorf tube with four ZrO_2 milling balls (350 mg in total mass) at 25–30 Hz for 3 h (see Figure S1). Similar product distribution was obtained when a mixture of **1c** (0.59 mol) and NFSI (1.0–2.0 equiv) was milled in a stainless steel milling jar (15 mL of internal volume) using one milling ball (4.0 g) of the same material. After the milling was halted, the crude reaction mixture was immediately analyzed by PXRD, IR and NMR. Isolation of **2c** and **2c''** was carried out by column chromatography on silica gel using mixtures of *n*-hexane and ethyl acetate as eluent (40:1 v/v to pure ethyl acetate).

2.2 Mechanochemical reaction of 3 with NFSI

A mixture of **3** (0.734 mmol) and NFSI (1.0 equiv) was milled in a stainless steel milling jar (15 mL of internal volume) using one stainless steel milling ball (4.0 g) of the same material at 30 Hz for 3 h. Isolation of **4a**, **4b**, and **4d** was carried out by column chromatography on silica gel using mixtures of *n*-hexane and ethyl acetate as eluent (20:1 to 1:1 v/v).

2.2 Mechanochemical reaction of 5 with NFSI

A mixture of **5** (0.367 mmol), NFSI (2.0 equiv), and K_2CO_3 (10 mol %) was milled in a stainless steel milling jar (15 mL of internal volume) using one stainless steel milling

ball (4.0 g) of the same material at 30 Hz for 3 h. The milling process was carried out in the absence of external heating or thermally assisted at 40 °C and 60 °C (Figure S2). Isolation of **6** was carried out by column chromatography on silica gel using mixtures of n-hexane and ethyl acetate as eluent (10:1 to 6:1 v/v).

3.0 Characterization of the products.

2-Fluoro-1,3,5-trimethoxybenzene (**2c**) [3].
^{1}H NMR ($CDCl_3$, 600 MHz): δ 6.16 (d, 2H, *J* = 5.9 Hz), 3.87 (s, 6H), 3.78 (s, 3H) ppm. ^{13}C{^{1}H} NMR ($CDCl_3$, 150 MHz): δ 155.7 (d, *J* = 3.2 Hz), 148.8 (d, *J* = 9.2 Hz), 137.9 (d, *J* = 236.7 Hz), 92.4, 56.7, 55.8 ppm. ^{19}F NMR ($CDCl_3$, 564 MHz): δ -168.39 (t, *J* = 5.9 Hz) ppm; (2,4-difluoro-1,3,5-trimethoxybenzene δ -160.37 (d, *J* = 7.3 Hz) ppm).

4,4-Difluoro-3,5-dimethoxy-2,5-cyclohexadienone (**2c''**) [4].
^{1}H NMR ($CDCl_3$, 600 MHz): δ 5.50 (t, 2H, *J* = 2.3 Hz), 3.84 (s, 6H) ppm. ^{13}C{^{1}H} NMR ($CDCl_3$, 150 MHz): δ 184.7, 161.2 (t, *J* = 22.1 Hz), 106.6 (t, *J* = 241.3 Hz), 102.6 (t, *J* = 3.5 Hz), 56.9 ppm. ^{19}F NMR ($CDCl_3$, 564 MHz): δ -112.49 ppm.

1-(Phenylsulfonyl)-1*H*-imidazole (**4a**) [5].
^{1}H NMR ($CDCl_3$, 600 MHz): δ 8.02 (s, 1H), 7.95 (d, 2H, *J* = 8.0 Hz), 7.69 (t, 1H, *J* = 7.5 Hz), 7.58 (t, 2H, *J* = 7.6 Hz), 7.31 (s, 1H), 7.10 (s, 1H) ppm. ^{13}C{^{1}H} NMR ($CDCl_3$, 150 MHz): δ 138.0, 136.8, 135.0, 131.6, 130.0, 127.4, 117.6 ppm.

2-Methyl-1-(phenylsulfonyl)-1*H*-imidazole (**4c**) [5].
^{1}H NMR ($CDCl_3$, 600 MHz): δ 7.89 (d, 2H, *J* = 7.6 Hz), 7.69 (t, 1H, *J* = 7.5 Hz), 7.58 (t, 2H, *J* = 7.7 Hz), 7.43 (d, 1H, *J* = 2.0 Hz), 6.91 (d, 1H, *J* = 1.6 Hz), 2.52 (s, 3H) ppm. ^{13}C{^{1}H} NMR ($CDCl_3$, 150 MHz): δ 146.0, 138.0, 134.8, 129.8, 128.1, 127.4, 119.5, 15.2 ppm.

1-(Phenylsulfonyl)-1*H*-benzo[*d*]imidazole (**4d**) [5].

^{1}H NMR ($CDCl_3$, 600 MHz): δ 8.39 (s, 1H), 8.00 (d, 2H, *J* = 7.6 Hz), 7.87 (d, 1H, *J* = 7.9 Hz), 7.77 (d, 1H, *J* = 7.9 Hz), 7.63 (t, 1H, *J* = 7.6 Hz), 7.53 (t, 2H, *J* = 7.9 Hz), 7.42-7.34 (m, 2H) ppm. ^{13}C{^{1}H} NMR ($CDCl_3$, 150 MHz): δ 144.2, 141.3. 137.7, 134.9, 130.9, 129.9, 127.3, 125.8, 125.0, 121.3, 112.6 ppm.

N-(1-Acetyl-1*H*-indol-3-yl)-*N*-(phenylsulfonyl)benzenesulfonamide (**6**) [6].

^{1}H NMR ($CDCl_3$, 600 MHz): δ 8.39 (d, 1H, *J* = 8.6 Hz), 8.00 (dd, 4H, *J* = 8.5, 1.6 Hz), 7.70-7.66 (m, 2H), 7.55-7.51 (m, 4H), 7.36-7.31 (m, 1H), 7.29 (s, 1H) 7.15-7.11 (m, 1H), 6.93 (d, 1H, 2H, *J* = 8.0 Hz), 2.57 (s, 3H) ppm. ^{13}C{^{1}H} NMR ($CDCl_3$, 150 MHz): δ 168.3, 139.1, 134.5, 134.2, 129.1, 128.7, 127.7, 127.0, 126.1, 124.3, 118.8, 116.7, 116.6, 23.9 ppm.

4.0 Copies of the NMR spectra

^{1}H NMR spectrum of 2-fluoro-1,3,5-trimethoxybenzene (**2c**)

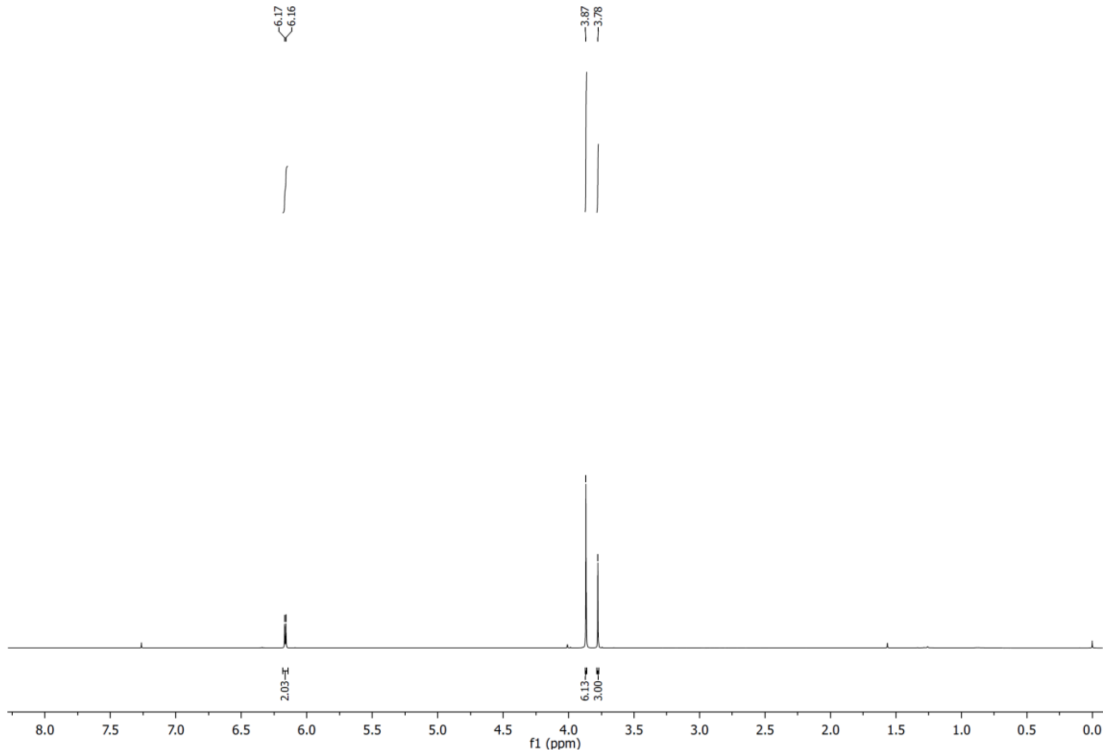

$^{13}C\{^{1}H\}$ NMR spectrum of 2-fluoro-1,3,5-trimethoxybenzene (**2c**)

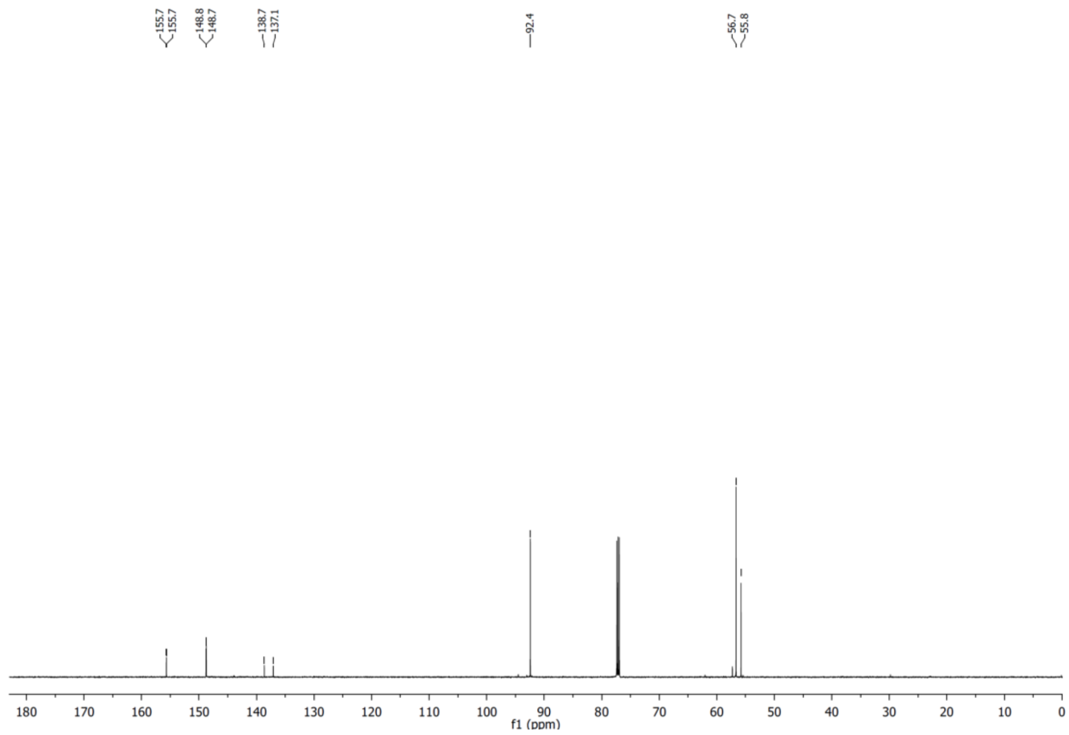

^{19}F NMR spectrum of 2-fluoro-1,3,5-trimethoxybenzene (**2c**)

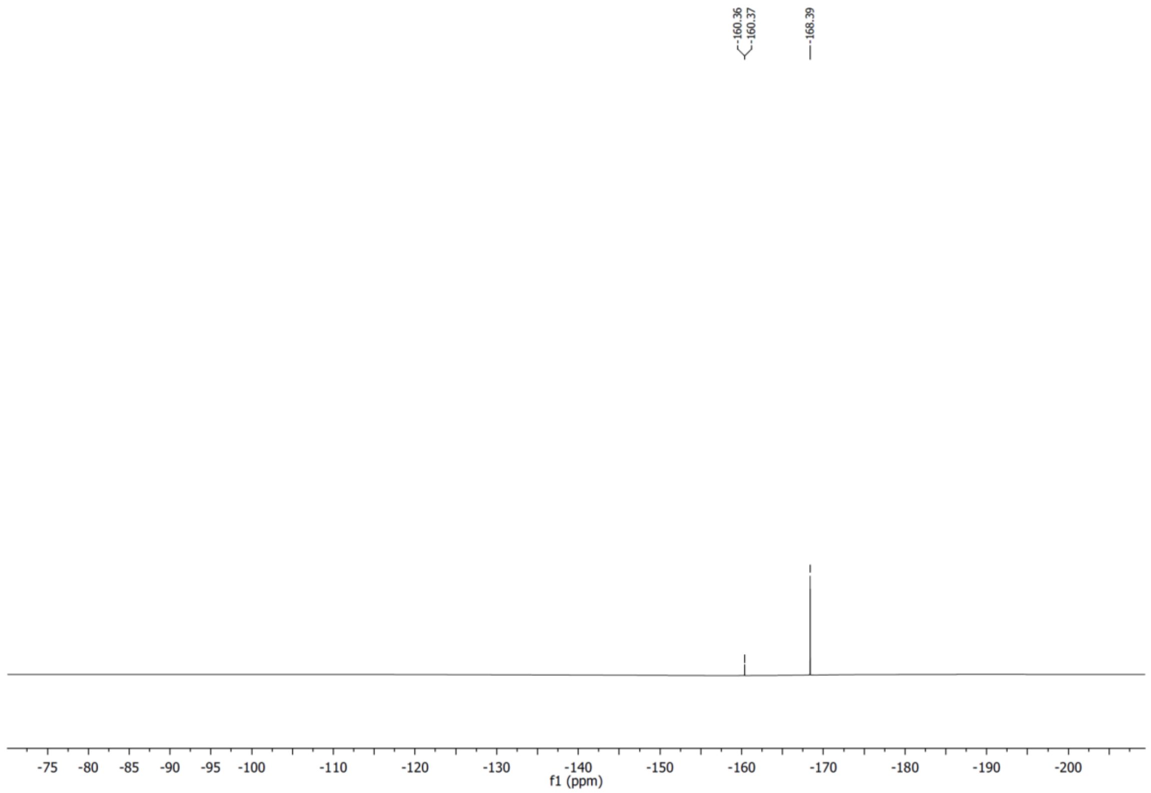

^{1}H NMR spectrum of 4,4-difluoro-3,5-dimethoxy-2,5-cyclohexadienone (**2c''**)

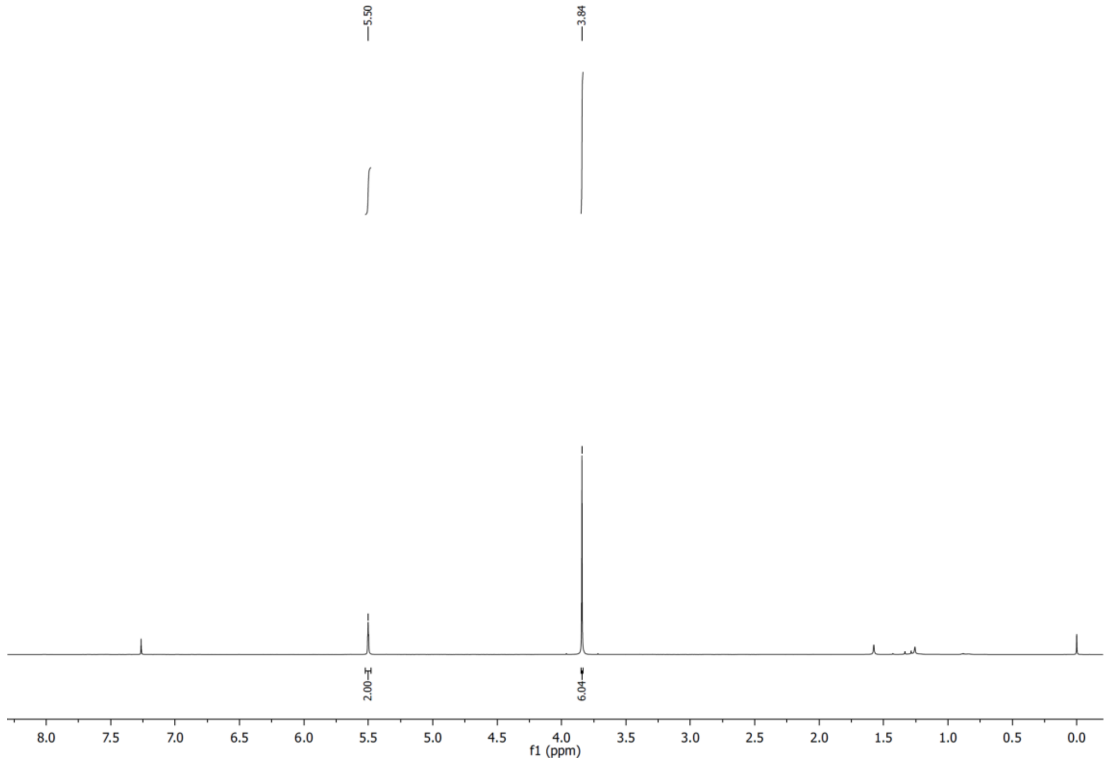

$^{13}C\{^{1}H\}$ NMR spectrum of 4,4-difluoro-3,5-dimethoxy-2,5-cyclohexadienone (**2c''**)

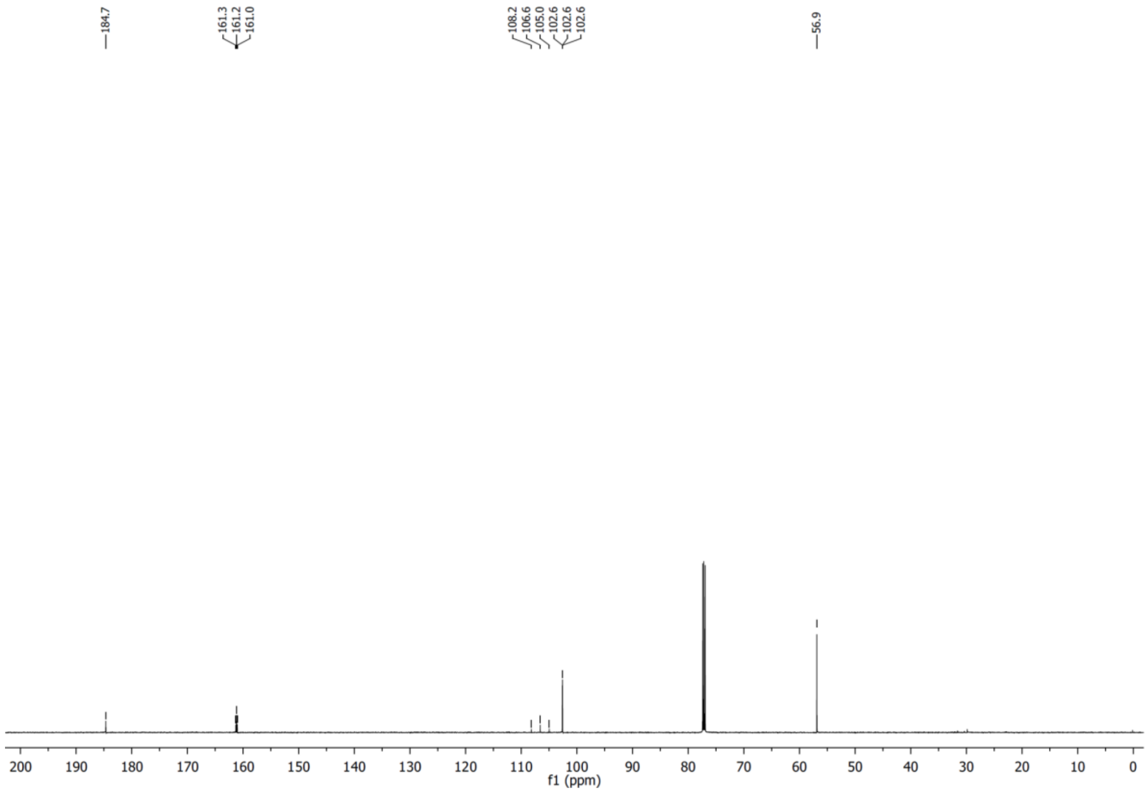

^{19}F NMR spectrum of 4,4-difluoro-3,5-dimethoxy-2,5-cyclohexadienone (**2c''**)

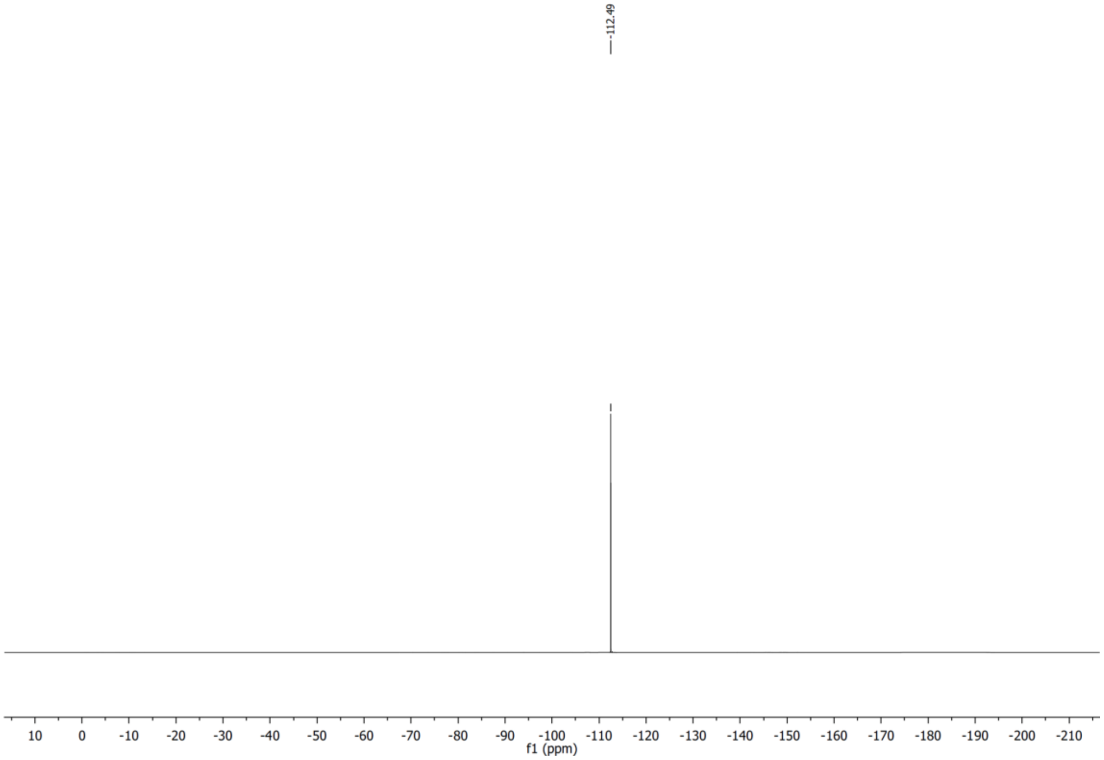

^{1}H NMR spectrum of 1-(phenylsulfonyl)-1H-imidazole (**4a**)

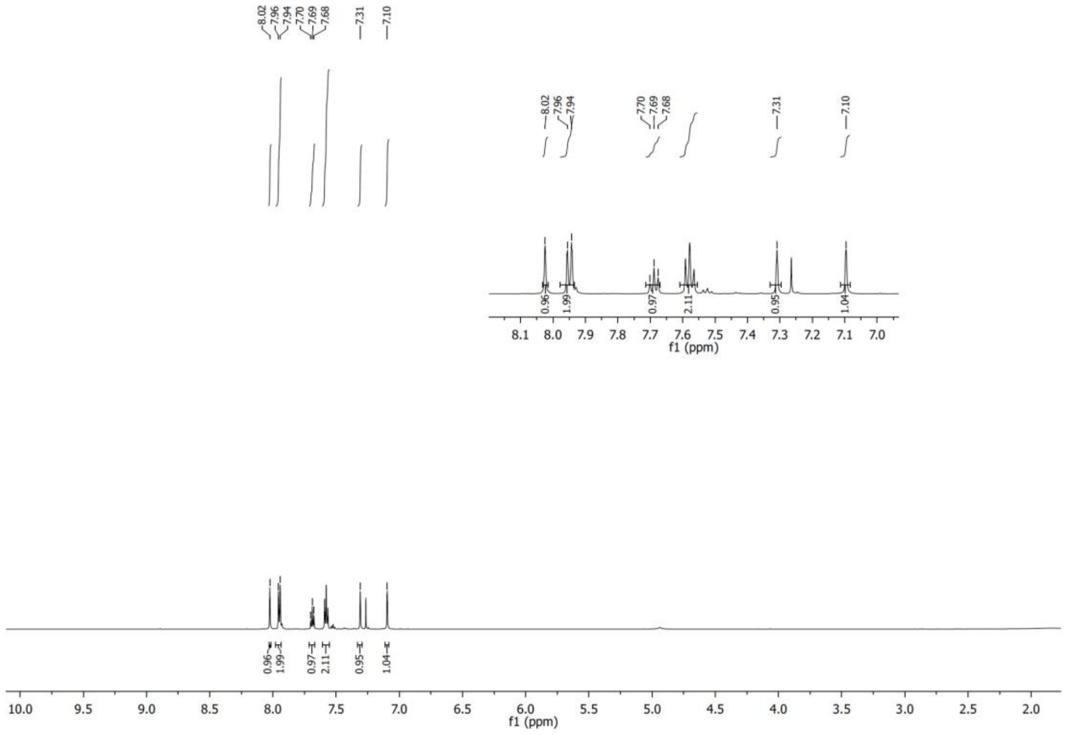

^{13}C{^{1}H} NMR spectrum of 1-(phenylsulfonyl)-1H-imidazole (**4a**)

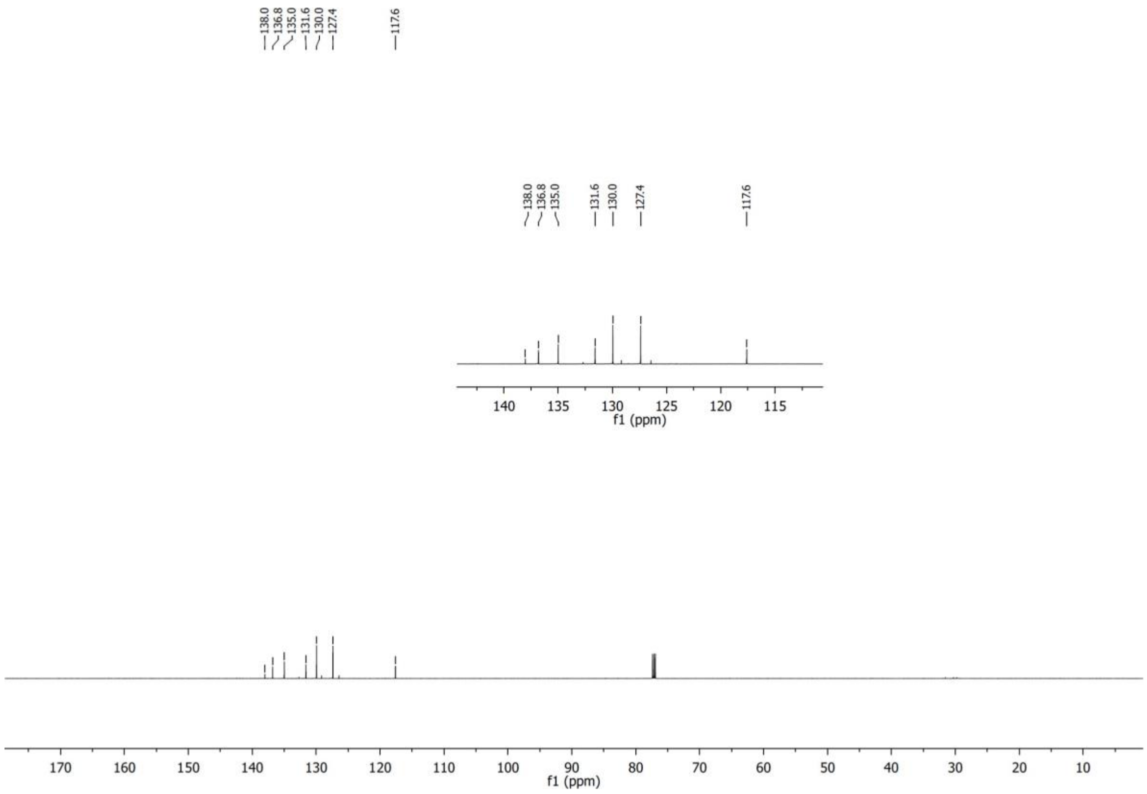

^{1}H NMR spectrum of 2-methyl-1-(phenylsulfonyl)-1*H*-imidazole (**4c**)

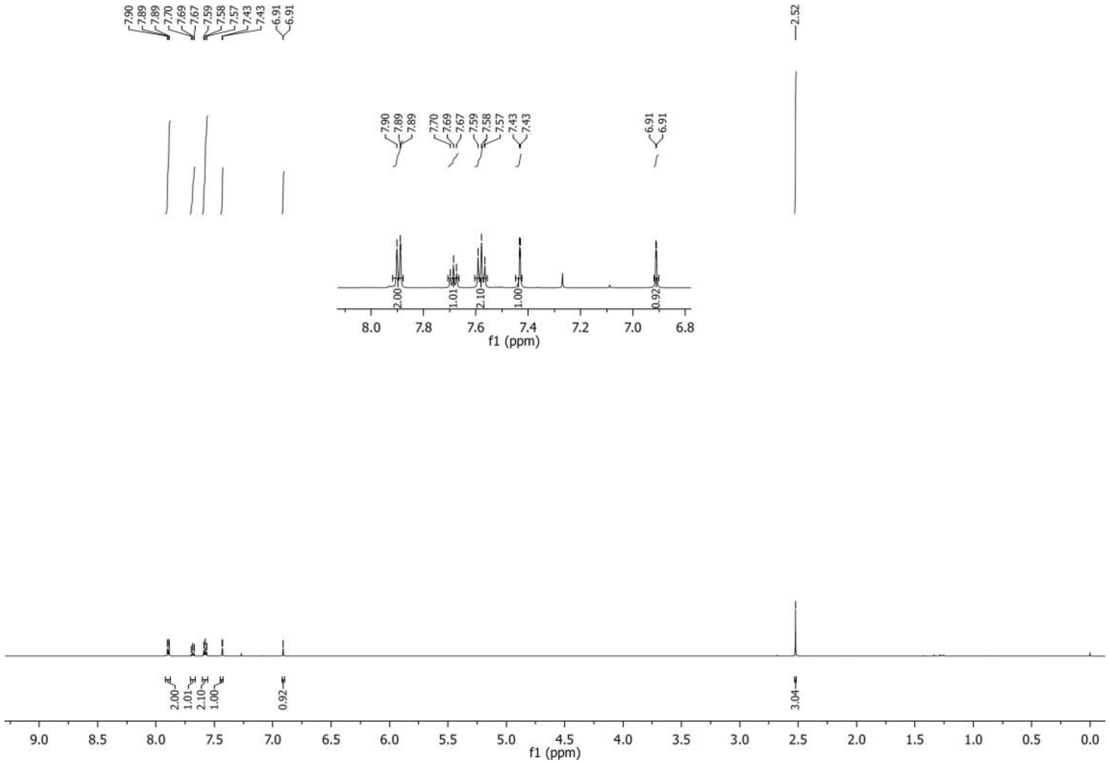

^{13}C{^{1}H} NMR spectrum of 2-methyl-1-(phenylsulfonyl)-1*H*-imidazole (**4c**)

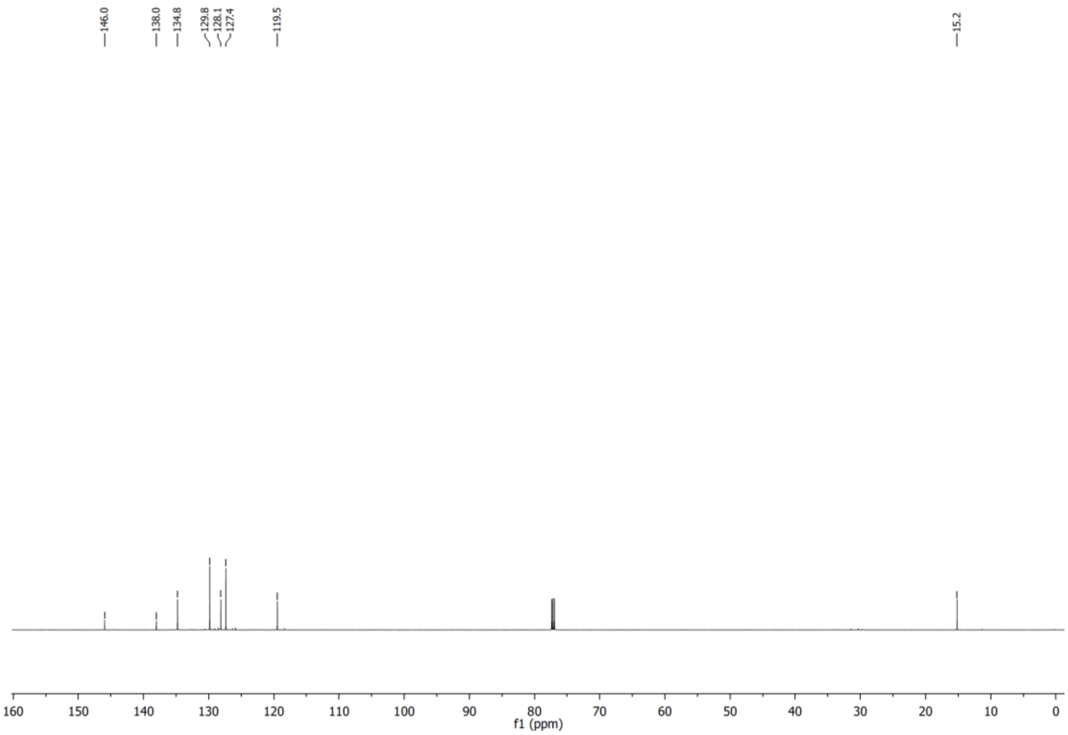

^{1}H NMR spectrum of 1-(phenylsulfonyl)-1*H*-benzo[*d*]imidazole (**4d**)

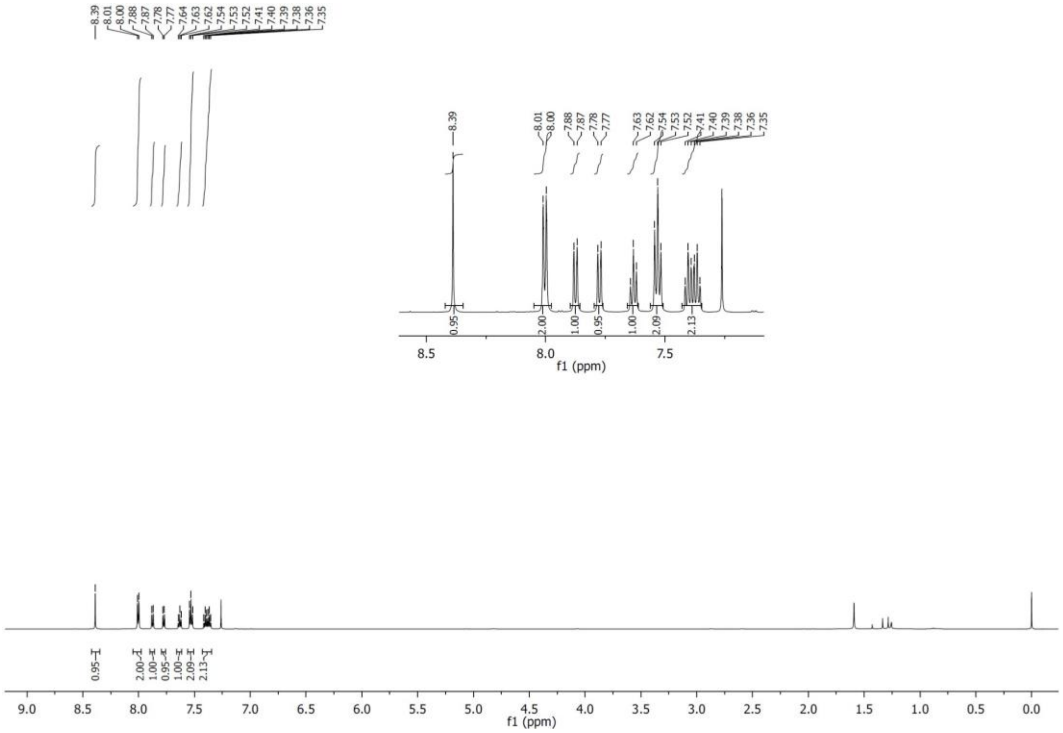

^{13}C{^{1}H} NMR spectrum of 1-(phenylsulfonyl)-1*H*-benzo[*d*]imidazole (**4d**)

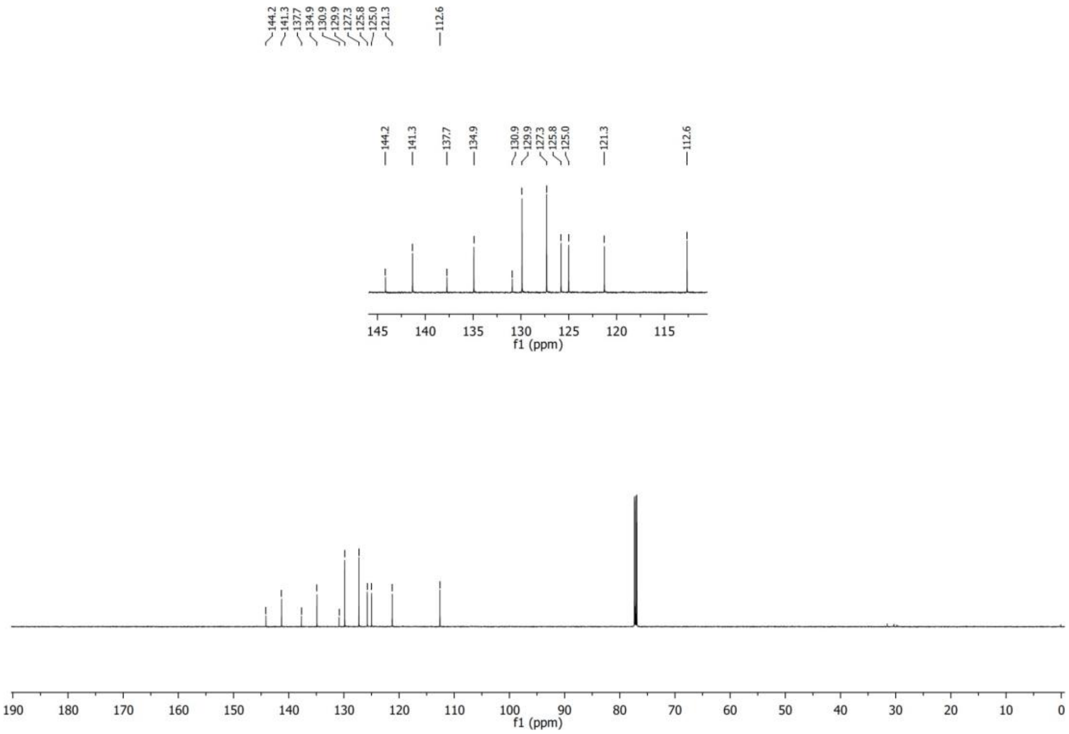

^{1}H NMR spectrum of indole derivative **6**

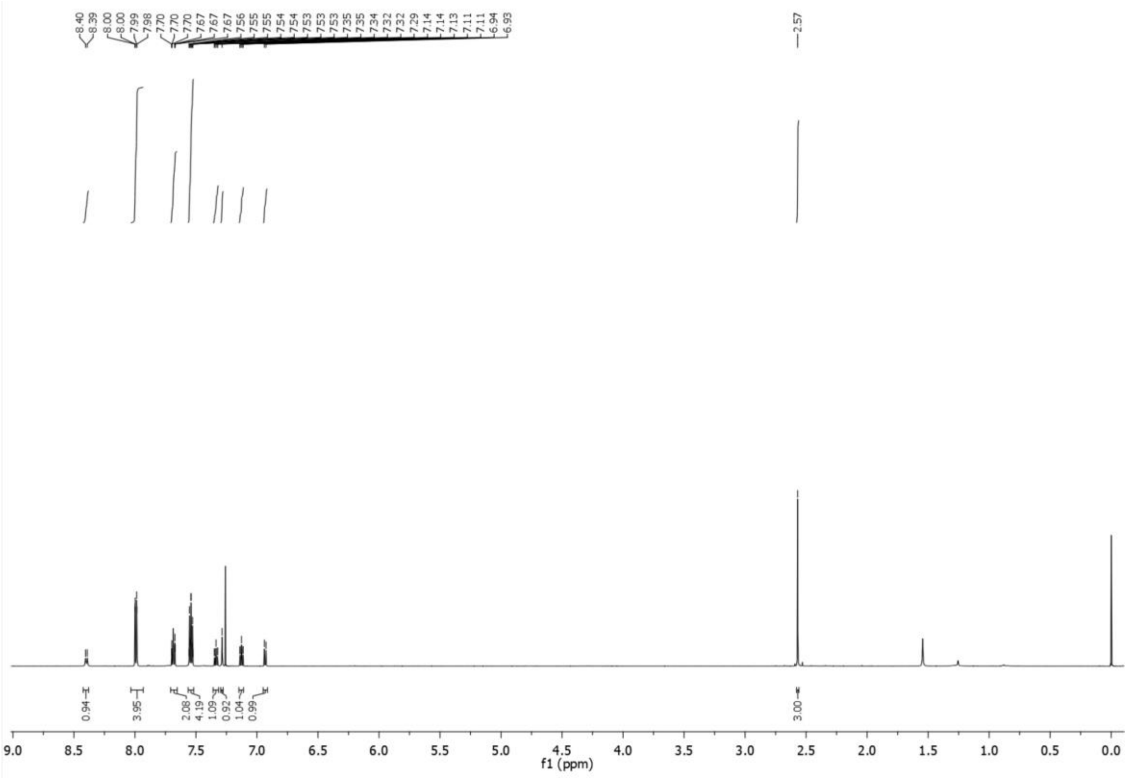

^{13}C{^{1}H} NMR spectrum of indole derivative **6**

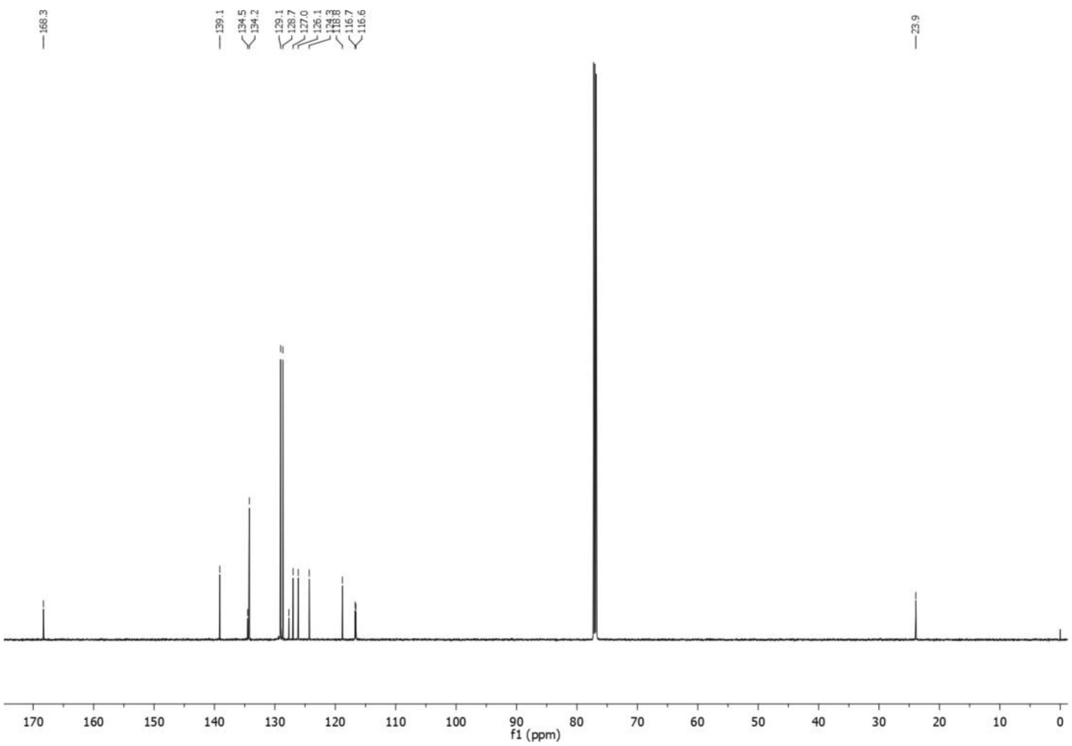

5.0 References

1. Medina-Mercado, I.; Asomoza-Solís, E. O.; Martínez-González, E.; Ugaide-Saldívar, V. M.; Ledesma-Olvera, L. G.; Barquera-Lozada, J. E.; Gómez-Vidales, V.; Barroso-Flores, J.; Frontana-Uribe, B. A.; Porcel. S. *Chem. Eur. J.* **2020**, *26*, 634–642. doi:10.1002/chem.201904413

2. Lukin, S.; Užarević, K.; Halasz, I. *Nature Protocols* **2021**, *16*, 3492−3521. doi:10.1038/s41596-021-00545-x

3. Bora, P. P.; Bihani, M.; Plummer, S.; Gallou, F.; Handa, S. *ChemSusChem* **2019**, *12*, 3037–3042. doi:10.1002/cssc.201900316

4. Pavlinac, J.; Zupan, M.; Stavber, S. *Molecules* **2009**, *14*, 2394–2409. doi:10.3390/molecules14072394

5. Jie, K.; Wang, Y.; Huang, L.; Guo, S.; Cai, H. *J. Sulfur Chem.* **2018**, *39*, 465–471. doi:10.1080/17415993.2018.1480725

6. Liu, H.-H.; Wang, Y.; Deng, G.; Yang, L. *Adv. Synth. Cat.* **2013**, *355*, 3369−3374. doi:10.1002/adsc.201300767
